# Impacts of heat stress on growth performance and its mitigation in small ruminants

**DOI:** 10.1093/af/vfaf021

**Published:** 2025-08-06

**Authors:** Silpa Mullakkalparambil Velayudhan, Ilavarasi Gunasekaran, Balamurugan Narasingam, Agnishwaran Ramajothi, Vanmathi Arulselvam, Eldhose Dona Mary, Darwin Ernest Angelin Shyona, Kalaignazhal Gajendirane, Rebez Ebenezer Binuni, Sejian Veerasamy

**Affiliations:** Rajiv Gandhi Institute of Veterinary Education and Research, Kurumbapet, Puducherry 605009, India; Rajiv Gandhi Institute of Veterinary Education and Research, Kurumbapet, Puducherry 605009, India; Rajiv Gandhi Institute of Veterinary Education and Research, Kurumbapet, Puducherry 605009, India; Rajiv Gandhi Institute of Veterinary Education and Research, Kurumbapet, Puducherry 605009, India; College of Veterinary Science and Animal Husbandry, Odisha University of Agriculture and Technology, Bhubaneshwar, Odisha 751003, India; College of Veterinary and Animal Sciences, Kerala Veterinary and Animal Sciences University Pookode, Wayanad, Kerala 673576, India; West Bengal University of Animal and Fishery Sciences, Kolkata, West Bengal 700037, India; College of Veterinary Science and Animal Husbandry, Odisha University of Agriculture and Technology, Bhubaneshwar, Odisha 751003, India; Rajiv Gandhi Institute of Veterinary Education and Research, Kurumbapet, Puducherry 605009, India; Centre for Climate Resilient Animal Adaptation Studies, ICAR-National Institute of Animal Nutrition and Physiology, Adugodi, Bangalore 560030, India; Rajiv Gandhi Institute of Veterinary Education and Research, Kurumbapet, Puducherry 605009, India

**Keywords:** climate change, goat, growth, heat stress, sheep, small ruminants

**Implications**

• Climate change and the associated stressors, particularly heat stress is a serious challenge for livestock producers, globally. Even though small ruminants are considered as ideal species with better adaptability to altering environmental conditions, these species also face detrimental effects on growth due to heat stress
• Growth, a crucial trait gets disrupted in heat-stressed animals and is reflected by alterations in hormonal status (growth hormone (GH), insulin-like growth factor-1 (IGF-1), thyroxin (T4) and triiodothyronine (T3), adrenocorticotropic (ACTH), thyroid stimulating hormone, follicle-stimulating (FSH), luteinizing hormones (LH), prolactin and leptin) and molecular dynamics (*GH*, *leptin* (*LEP*), *growth hormone receptor* (*GHR*), *IGF-1*, *myosin enhancer factor* (*MEF2B*), *myogenic factor* (*MYF5*), and *myostatin* (*MSTN*))
• Conventional growth evaluation metrics can be integrated to artificial intelligence (AI) tools to evaluate growth performance. Effective adoption of advanced AI tools with genomic approaches might unravel molecular mechanisms associated with thermal insult-induced alteration in growth traits.

## Introduction

Small ruminants have been amongst the first farm animals to be domesticated by humans. Primarily, these animals are raised for meat, milk and skin ensuring global food security in addition to providing draught power and financial services. Small ruminants are highly valued for their versatility and excellent adaptability to varied geographical and climatic conditions as evident from their wide distribution pattern in Asian and African continents with rough climatic conditions.

The term “climate change” can be stated as the significant changes in climate over decades or longer. As per the Intergovernmental Panel on Climate Change (IPCC) report, global average surface temperature has been predicted to increase between 1.8 and 4.0°C (IPCC, 2022), while an increase in average global temperatures of 1.5 to 2.5°C is projected to subject 20-30 % of plant and animal species to risk of extinction (FAO, 2007). Therefore, climate change is a serious global concern demanding coordinated effort from farmers, researchers and policy makers to effectively combat its adverse effects.

A significant manifestation of this climate crisis is heat stress in livestock. It is the sum of external and internal heat sources that act upon an animal, causing an increase in body temperature. Heat stress impairs growth performance, milk production, reproductive performance, meat production and immune status in animals, varying among species, breed, age and production level. The magnitude of adverse impacts of heat stress on the animal determines its adaptive potential. When compared to other livestock species, small ruminants possess better traits for adaptation in harsh environmental conditions, including behavioral, morphological, physiological, endocrine, molecular and cellular characteristics, many of which are genetic.

**Figure 1.**
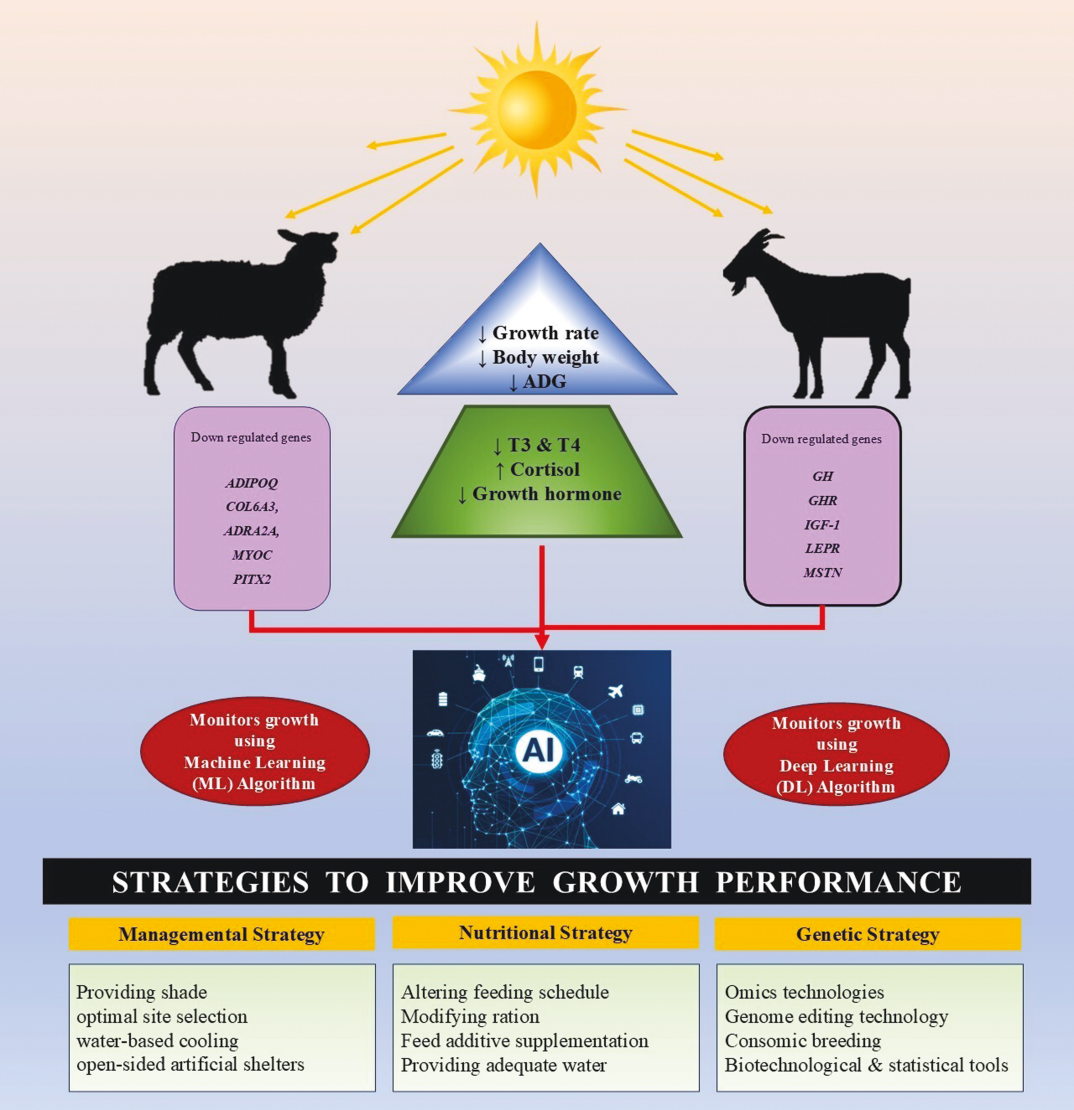
Graphical abstract.

The advancing biotechnological and bioinformatic approaches have enabled improved comprehension of the molecular basis of biological processes in livestock. Although, these technologies have been employed to assess production outcomes, comparatively only limited studies have explored their application in understanding heat stress response. Heat stress associated molecular responses related to growth traits in small ruminants are relatively less documented. Thus, this article provides snippets on heat stress impacts on growth-related traits in small ruminants and provides an overview of methods to quantify such responses using artificial intelligence (AI). Further, the article collates possible mitigation strategies to combat adverse effects of heat stress in small ruminants. Figure 1 presents the graphical abstract of the article.

## Significance of Small Ruminants

Globally, since time immemorial, small ruminant rearing has been practiced and has long been a sustainable livelihood resource among rural stratum. Small ruminant production ensures food and economic security of marginal farmers, especially in developing countries. Goats are often seen as an alternative to cows, particularly by farmers with limited financial resources, as they provide long-term economic return. Goats in Asia presently account for 57.7% of global goat population. Meanwhile, Africa raises around 35.7% of global goat population. India has world’s largest goat population, totaling 150 million, while China has world’s second-largest goat population of 132.4 million (Goat Population by Country 2024).

China has world’s largest sheep population, with 194 million sheep, or 14.69% of the global total. India and Australia holds 75.3 million and 70.2 million respectively. Iran has 55.6 million sheep and a 4.21% share (Sheep Population by Country 2024). Global sheep meat production exceeds 9 million tons per year, with developing countries leading the way. Sheep meat consumption ranks fourth behind pork, chicken, and beef meat. Sheep and goats provide around 20.8% of dairy products, accounting for 1.3% and 1.9% of global milk production, respectively (Mazinani and Rude, 2020).

The above statistics clearly emphasizes the distribution and production statistics of sheep and goat farming across the globe. Amidst the challenging climate, small ruminant production has been a vital component of both food and economic security, particularly in resource-limited and marginal agricultural regions. In the present phase of rising global population, increasing demands, decreasing resources and climate change, small ruminant rearing can be projected to be significant as they possess immense potential to ensure sustainability.

## Different Factors influencing Growth Performance in Small Ruminants

Growth is a basic measure of production in livestock and is influenced by genetic, environmental and management aspects. It is crucial to understand these factors to sustain livestock farming and increase productivity. On that note, heat stress is one of the critical factors affecting growth status in farm animals. It leads to decreased feed intake and increased water intake, leading to reduced growth performance. Further, nutrition is regarded to play a decisive role in optimizing growth performance. Additionally, inadequate management practices like poor parasite control, improper housing and farm hygiene will further compromise animal growth.

**Figure 2.**
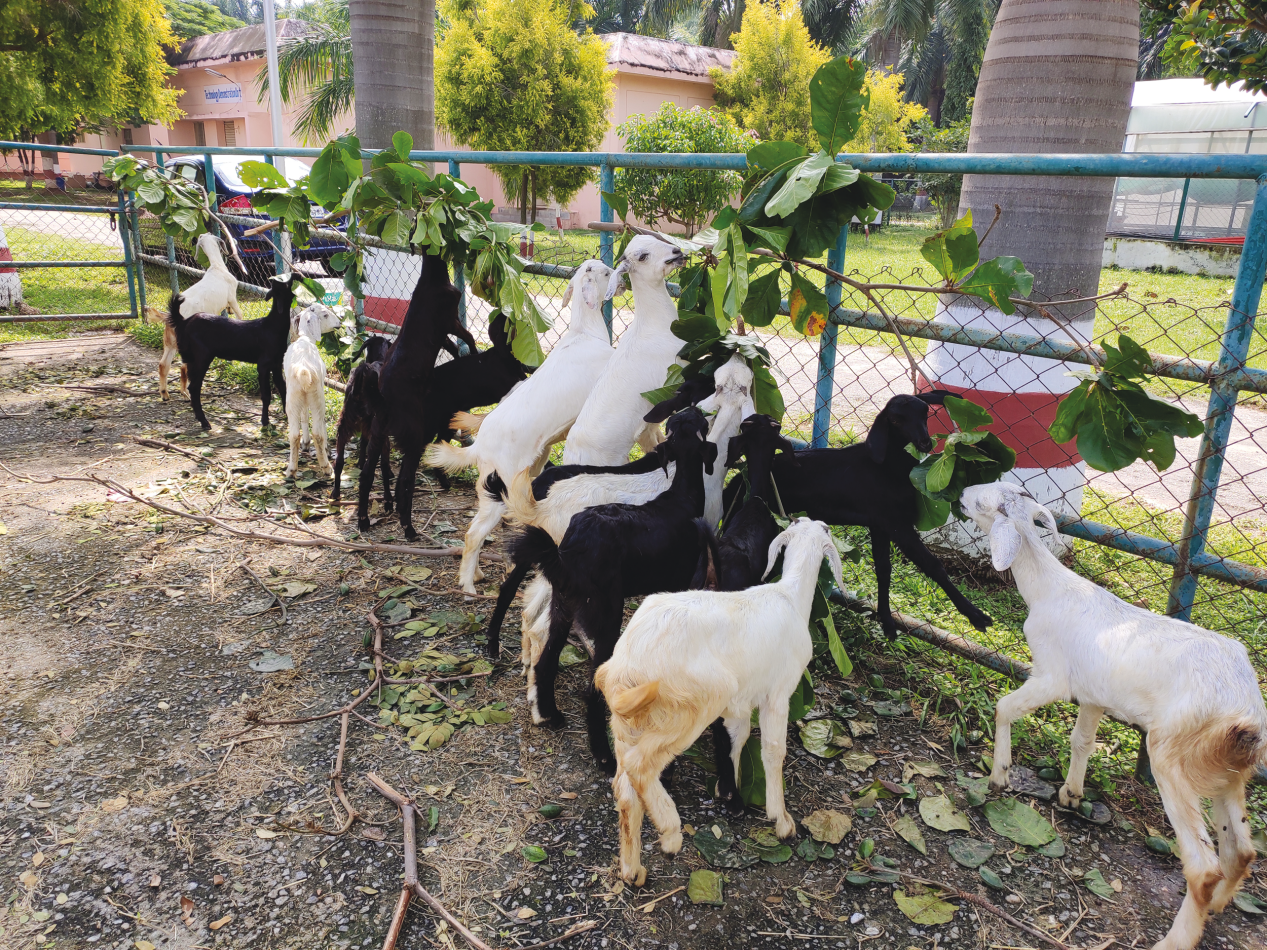
Feeding management of two indigenous breeds of goats.

Genetics also plays a significant role in the growth performance of small ruminants as certain breeds are selectively bred for traits like faster growth rates and or larger body size. Selective breeding techniques have shown the capacity to improve economically significant features including body weight at weaning and adult size. Therefore, a holistic approach involving proper nutrition, breeding practice, environmental management, and animal welfare is essential for sustaining optimal growth performance in small ruminants. Figure 2 describes the feeding management of two indigenous Nandidurga and Bidri goat breeds to ensure appropriate growth performance of these animals.

## Heat Stress as the Most Important Factor Negatively Influencing Growth in Small Ruminants

In tropical and subtropical regions, heat stress has been demonstrated to adversely affect livestock growth and performance. Body weight, average daily gain, growth rate, feed intake, production efficiency, or weight gain per unit of feed energy are all significantly impacted by heat stress (Rebez et al., 2024). Heat stress associated fodder scarcity is of a serious concern as shortage of feed resources due to altered temperatures act as a stressor that affects growth. Alterations in biological processes, including disruptions in metabolism of protein, water, energy and minerals, hinder growth of animals in tropical and subtropical areas.

Heat stress also impairs growth-related organs (pituitary, thyroid, liver, muscle, thymus and hypothalamus) and disrupts growth performance by regulating appetite control center and digestive enzyme activity, leading to reduced food intake and digestive functions in livestock (Pragna et al., 2018). Reduced anabolic activity and increased tissue catabolism, mostly in fat depots and/or lean body mass, along with lower thyroid hormone concentrations, decreased feed intake, and increased tissue damage, are the consequence of heat stress on growth performance of ruminants.

Further, prolonged heat stress causes systemic inflammation, which can be observed by elevated levels concentrations of circulating leukocytes and cytokines, leading to hyperventilation, decreased growth performance, and weakened health. This increased inflammatory tone is unfavorable for muscle growth as cytokines reduce myoblasts’ ability to appropriately promote muscle fiber hypertrophy. Furthermore, since more nutrients must be redistributed for homeostatic processes, inflammation lowers metabolic efficiency. Therefore, these detrimental effects of heat stress can reduce growth performance in small ruminants.

## Impact of Heat Stress on Growth Performance of Sheep

Exposure to thermal stress disrupts metabolic and digestive functions, attributed to changes in feeding patterns and reduced nutrient absorption efficiency. Further, heat stress can compromise dry matter intake, triggering decreased appetite and feeding aversion. Heat stress has debilitating consequences on sheep growth and is manifested as reduced growth rate, average daily feed intake, impaired daily weight gain, and compromised overall live weight (Zhang et al., 2024).

The duration and timing of heat stress during gestation also plays a critical role in determining the extent of fetal growth restriction, with extended periods of heat stress leading to more pronounced adverse effects. For instance, prolonged exposure to heat stress during pregnancy can severely impede fetal growth in ewes, resulting in significantly reduced birth weights. Heat stress is thus a significant factor that profoundly impacts sheep growth performance, primarily by reducing feed intake, weight gain, and overall productivity.

## Impact of Heat Stress on the Growth Performance of Goat

Goats exhibit variations in physiological attributes and growth patterns across diverse thermal comfort zones. Heat stress in goats result in substantial weight loss, decreased feed consumption, and reduced average daily gain (Pragna et al., 2018) due to decreased feed consumption at high temperatures.

**Figure 3.**
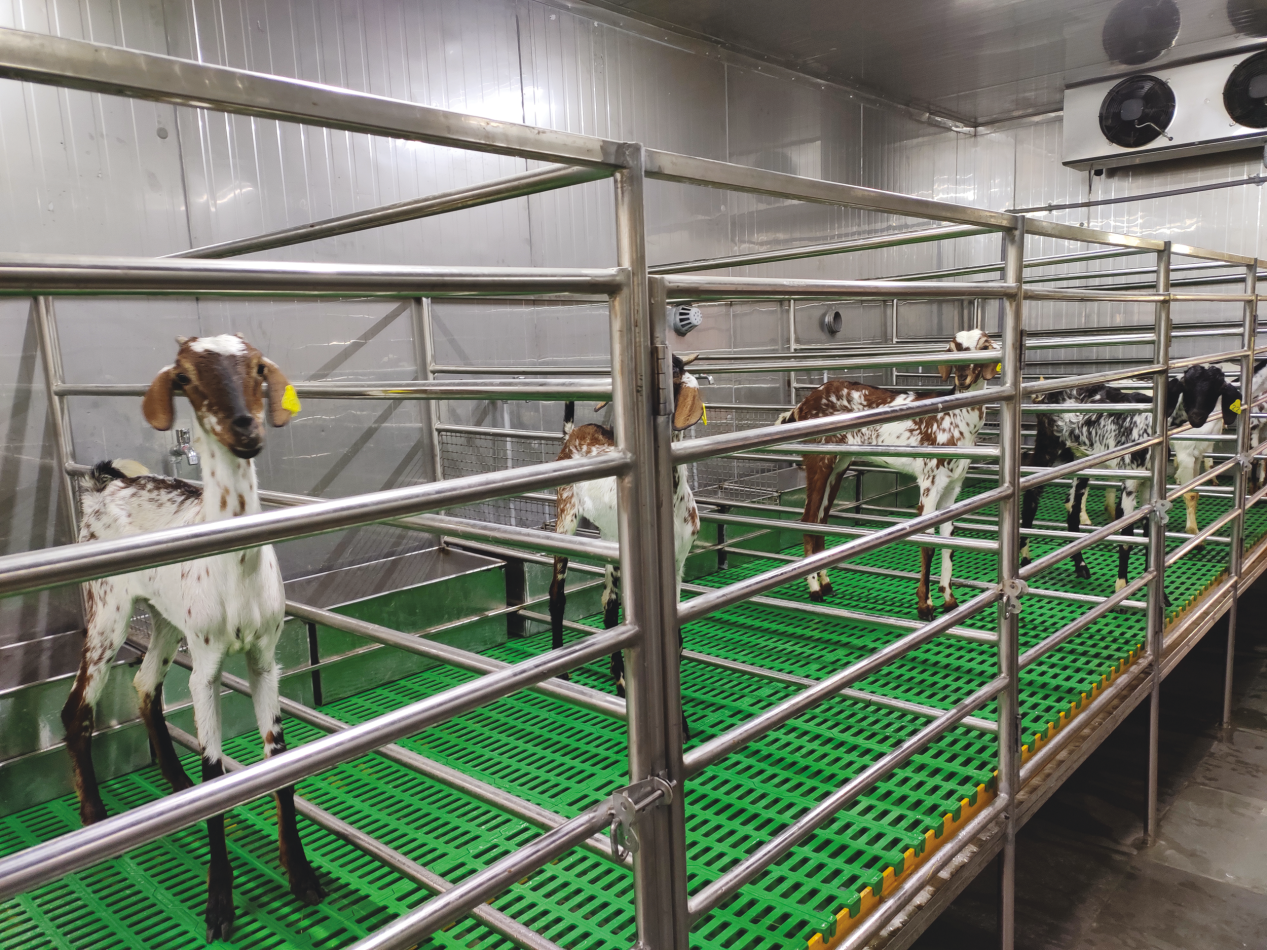
Kodi Aadu goat kept in climate chamber for assessing heat stress impact on growth performance.

Acute heat stress conditions typically leads to substantial reductions in body weight, resulting in notable weight losses of up to 0.5 kg/°C per animal, making it a high concern as body weight is a critical economic trait in goat production. Thus, the relationship between heat stress and diminished goat productivity is undoubtedly significant. Elevated temperature triggers substantial decline in growth performance, emphasizing the urgency for targeted mitigation strategies. Figure 3 describes the Kodi Aadu goats kept in climate chamber for studying the heat stress impact on their growth performance.

## Impact of Heat Stress on Growth Governing Endocrinology in Sheep

Heat stress alters the endocrine dynamics by reducing anabolic hormones responsible for promoting growth and development of muscles, and by increasing catabolic hormones, that catabolizes tissues and retards growth. On that note, heat stress decreases the concentration of thyroid hormones such as thyroxin (T4) and triiodothyronine (T3), affecting the basal metabolic rate essential for the process of growth (Tüfekci and Sejian, 2023).

Heat stress in small ruminants influences the cortisol levels. Increased levels of cortisol interferes catabolic process and also have an indirect impact on growth by affecting insulin-like growth factor-1 (IGF-1). Heat stress affects secretion of ghrelin which is vital for stimulation of growth hormone (GH) release. Further, GH production is affected by reduced feed intake in heat-stressed sheep that limits availability of key nutrients required for the synthesis of GH. In heat-stressed sheep, there is an increase in plasma GH concentration that can be attributed to reduced feed intake and reduced binding of GH to its receptor in heat-stressed animals (Sejian et al., 2013). Consequently, these alterations lead to diminished muscle development and thereby influence the meat quality and yield (Tüfekci and Sejian, 2023).

## Impact of Heat Stress on Growth Governing Endocrinology in Goat

The endocrine response in heat-stressed goats is nearly similar to that in sheep. The primary responses seen under heat stress are, decrease in anabolic and increase in catabolic hormones. Affected hormones include adrenocorticotropic (ACTH), thyroid stimulating hormone, GH, follicle-stimulating (FSH), luteinizing hormone (LH), as well as prolactin (PRL).

The hypothalamic–pituitary–thyroid (HPT) axis regulates energy expenditure by influencing basal metabolic rate through the actions of thyroid hormones. However, in an attempt to reduce heat load during prolonged heat stress, farm animals invariably exhibit decreased HPT activity. The negative effects of heat stress on thyroid activity and its associated decreased thyroid hormone levels are well established (Sejian et al., 2013). Further, farm animals also exhibit significant reduction in leptin levels under heat stress condition, attributing to reduced feed intake and conversion of all non-carbon sources into glucose as a part of behavioral and neuro-endocrine responses.

## Impact of Heat Stress on the Expression Patterns of Growth Governing Genes in Sheep and Goat

Heat stress has a profound effect on growth performance of animals by impairing physiological and metabolic pathways, subsequently altering the expression patterns of genes that govern growth. Numerous biological processes are regulated by these genes, including feed intake and utilization, cellular growth and differentiation, energy metabolism, muscle development, and fat deposition (Pragna et al., 2018). During heat stress, the expression of these genes is disrupted leading to a series of physiological alterations that impact overall growth performance. The impact of heat stress on the physiological growth pathways like muscular growth and development in small ruminants is associated with genes that encode GH, leptin (LEP), growth hormone receptor (GHR), insulin-like growth factor-1 (IGF-1), myosin enhancer factor (MEF2B), myogenic factor (MYF5), myostatin (MSTN), myogenin (MYOG), leptin receptor (LEPR), thyroid hormone receptor (THR), etc,. (Sejian et al., 2019; Ibrahim et al., 2023; Lu et al., 2023).

Heat stress lowers the expression of *GH, GHR* and *IGF-1* (Angel et al., 2018; Madhusoodhan et al., 2021) indicating compromised growth during heat stress. However, alteration in expression of these genes are noted across breeds, thus these genes serve as valuable indicators of growth performance, highlighting breed-specific adaptive responses to heat stress (Pragna et al., 2018). Additionally, genes such as *LEP* and *LEPR* involved in fat metabolism also show lower expression during heat stress (Angel et al., 2018). Further, the MSTN gene plays regulatory role in muscle development and differentiation, playing a critical role in meat production. However, a significant reduction in *MSTN* gene expression occurs in heat-stressed small ruminants (Abhijith et al., 2021; Spandan et al., 2022), typically influencing meat production.

In addition, genes such as *ADIPOQ*, *COL6A3*, *ADRA2A*, *MYOC*, and *PPARGC1A,* involved in muscle development and fat deposition are down regulated in heat-stressed small ruminants due to reduced body weight and average daily gain. (Zhang et al., 2024). Further, acute heat stress down regulates *PITX2* gene, a potent growth regulator (Li et al., 2019). A transcriptomic analysis reveals differentially expressed genes (DEGs) such as *ANGPT2*, *NPR1*, and *SLC13A5*, involved in fat metabolism under heat-stressed conditions (Lu et al., 2019). These findings suggest that alterations in specific gene expressions significantly influence growth performance under heat stress.

Heat stress affects myoblast proliferation and differentiation, ultimately leading to reduced growth in animals. The molecular basis of these processes can be assessed by identifying the expression of *PAX7, MYHC, MYOD, MYOG* and *MYF5* genes (Lu et al., 2023). Additionally, PCR-DNA sequencing has identified SNPs in genes such as *CAST*, *MYLK4*, *LEP*, *STAT5A*, *MEF2B*, and *TRPV1* in lambs that suggest a significant association between SNPs and traits related to growth performance and heat tolerance, indicating their potential role in enhancing the growth characteristics in the lambs (Ibrahim et al., 2023). Further, genes like *AGPAT2*, *MFAP32*, *ABCD2*, *SCD*, *SIRT3*, *RNF121*, and *YTHDC1* involved in intramuscular fat deposition could serve as potential candidate genes for improving production under heat stress (Gaspa et al., 2024). Table 1 provides an overview of the discussed genes that were influenced by heat stress in sheep and goats.

**Table 1.** List of genes altered in heat-stressed sheep and goats and their associated functions

Species	Breed	Genes	Pathways/ functions	Reference
Goat	Malabari goat	*GH, IGF-1, LEP, GHR, LEPR*	Cell growth, cell multiplication and differentiation, regulation of body weight	Angel et al., 2018
	Salem black	*GHR, IGF-1*	Cell growth, cell multiplication and differentiation	Madhusoodan et al., 2021
	Osmanabadi, Malabari and Salem Black	*IGF-1*	Cell growth, cell multiplication and differentiation	Pragna et al., 2018
	Malabari goat	*MSTN*	Muscular growth and development	Abhijith etal., 2021
	Kani aadu	*MSTN*	Muscular growth and development	Spandan et al., 2022
Sheep	Sarda ewes	*AGPAT2*, *MFAP32*, *ABCD 2*, *SCD*, *SIRT3*, *RNF121, YTHDC1*	Intramuscular fat deposition	Gaspa et al., 2024
	Hu sheep	*ADIPOQ*, *COL6A3*, *ADRA* *2A*, *MYOC*, *PPARGC1A*	Muscle development and fat deposition	Zhang et al., 2024
	Barki and Aboudeleik lambs	*CAST*, *MYLK4*, *LEP*, *STAT5A*, *MEF2B*, and *TRPV1*	Muscle growth and development	Ibrahim et al., 2023
	Hu sheep	PITX2	Growth and development	Li et al., 2019
	Hu sheep	*PAX7, MYHC, MYOD, MYOG, MYF5*	Myoblast proliferation and differentiation	Lu et al., 2023
	Hu lamb	*oar-miR-411a-5p*	Myoblast proliferation and differentiation	Lu et al., 2024
	Hu sheep	*ANGPT2, NPR1, SLC13A5*	Fat metabolism	Lu et al., 2019

**Figure 4.**
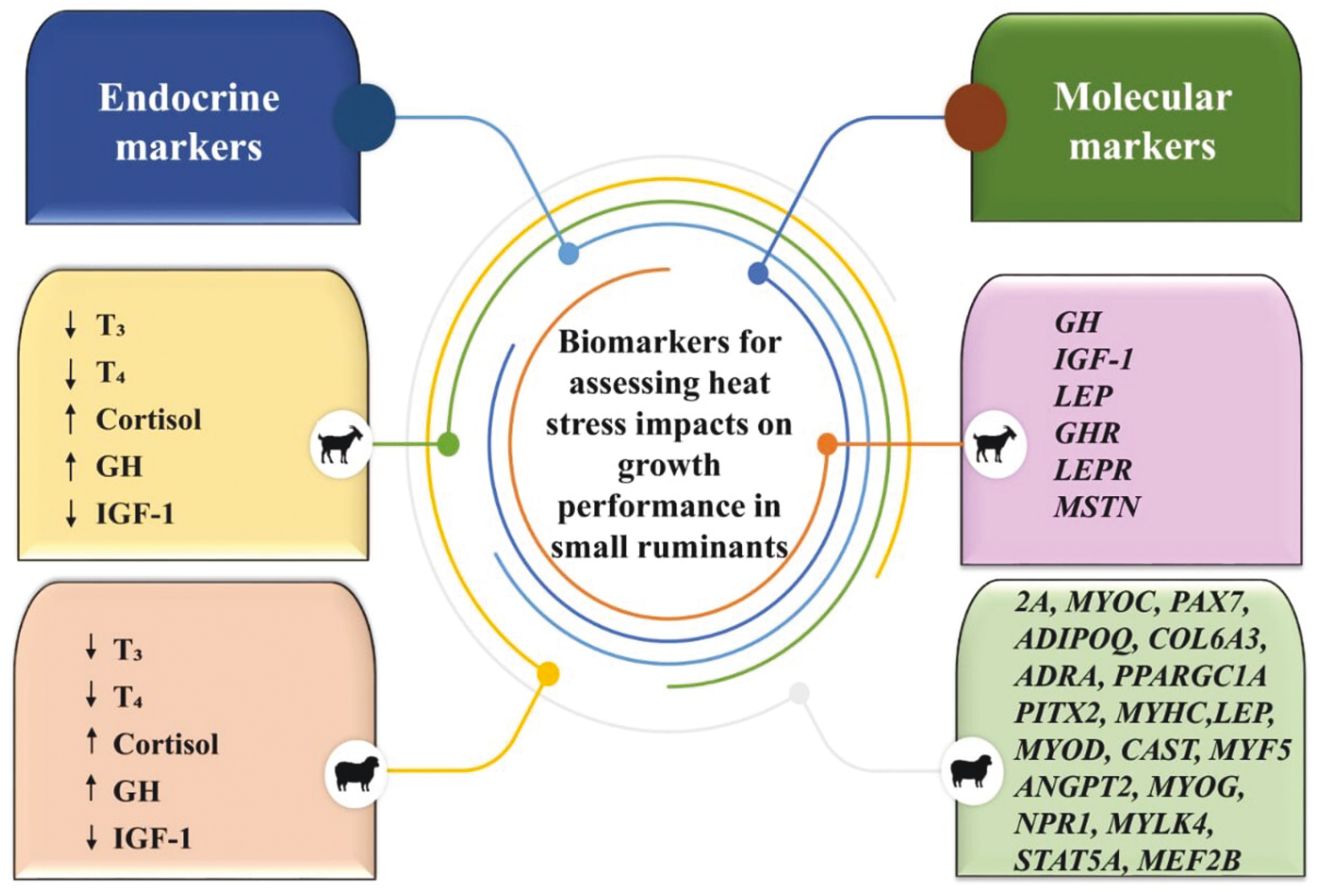
Biomarkers for assessing the impact of heat stress on growth performance in small ruminants. T_3_ – triiodothyronine, T_4-_ – thyroxine, GH – growth hormone, IGF-1 – insulin-like growth factor-1 as endocrine markers; GH – growth Hormone, IGF-1 – insulin-like growth factor-1, LEP – leptin, GHR – growth hormone receptor, LEPR – leptin receptor, MSTN – myostatin, 2A – actin alpha cardiac muscle 1, MYOC – myocilin, PAX7 – Paired box protein 7, ADIPOQ – adiponectin, C1Q and Collagen Domain Containing, COL6A3 – collagen type VI alpha 3 chain, ADRA – adrenoceptor alpha, PPARGC1A – peroxisome proliferator-activated receptor gamma coactivator 1 alpha, PITX2 – paired like homeodomain 2, MYHC – myosin heavy chain, LEP – leptin, MYOD – myogenic differentiation 1, CAST – calpastatin, MYF5 – myogenic factor 5, ANGPT2 – angiopoietin 2, MYOG – myogenin, NPR1 – Natriuretic Peptide Receptor 1, MYLK4 – Myosin Light Chain Kinase Family Member 4, STAT5A – Signal Transducer and Activator of Transcription 5A, and MEF2B – myocyte enhancer factor 2B, as molecular markers for assessing heat stress impacts on growth in small ruminants.

By altering the expression of genes related to thermoregulation, stress response, metabolism, protein synthesis, inflammation, and muscle development, heat stress impairs growth by reducing feed intake, muscle growth and energy efficiency, leading to reduced weight gain and productivity. Identifying specific genetic pathways disrupted by heat stress can enable breeders and researchers to pinpoint genetic markers linked to heat tolerance. This knowledge can be applied in breeding programs to develop heat-resilient animals with improved growth performance in tropical countries. Figure 4 summarizes the key biomarkers for assessing the impact of heat stress on growth performance in small ruminants.

## Methods to Measure Growth in Small Ruminants

Measuring growth rate facilitates evaluation of overall health, feed efficiency and productivity of animals, in addition to an indirect assessment of genetic potential, disease and stress factors affecting animal’s performance. Over the decades, several methods have been put forth by researches for measuring growth in small ruminants. These methods range from simple physical measurement of daily weight gain to artificial prediction models for measuring the growth in animals.

The foremost method of measuring growth in goat and sheep is body weight measurement (BW). The BW can be measured using weighing scale or weighing balance at fixed intervals and growth rate can be directly assessed using weight gain at that interval. The average daily gain (ADG) is the simplest and best method for measuring growth in sheep and goat. Further, linear body measurements can be used for computing growth in ruminants. These measurements include heart girth (HG), body length (BD), height at withers (HW), shoulder height (SH), hip width and cannon bone circumference. Three different prediction methods such as Shaffer’s formula, multiple regression and artificial neural network (ANN) have been reported for estimating the live weight in livestock species.

Body condition scoring (BCS) can act as an indirect method for depicting the growth. Animals with a moderate or high BCS are regarded to be in better growth rate, while low BCS in a growing animal indicates poor growth. Similarly, feed conversion ratio (FCR) has also been considered as an ancillary approach for measuring growth. Since, FCR is related to average daily gain (ADG) and average daily feed intake (ADFI), the increase in body mass per unit of feed can be ascertained to growth of an animal. Kleiber ratio (KR) is yet another growth measuring element in relation to ADG and metabolic body weight (MBW). It is termed as a valuable tool for determining growth efficiency and FCR and KR values are directly related to growth rate, with higher KR values indicating better growth rates.

Growth of an animal can be measured by quantifying growth-related biomarkers. The GH, T3, T4, IGF-1, IGF-2 and leptin are reported as the primary indicators for measuring growth potential. Similarly, the genes encoding *GH, GHR, THR, IGF-1, LEP,* and *LEPR* are identified as genes that facilitates release and site- specific action of those hormones. By measuring the levels of those hormones in serum and quantifying these genes for their expression could provide molecular evidence for assessing relative growth rate in animals. Thus, by employing any one or preferably a combination of the above discussed techniques the growth of sheep and goat can be measured to assesses their performances.

## Applications of Artificial Intelligence in Measuring Growth in Small Ruminants

The current technological bloom has accelerated production from climate-sensitive livestock sector and optimized production efficiency through enhanced adoption of cutting- edge AI tools. Adoption of advanced AI tools can improve the scope of assessment of growth in heat-stressed small ruminants. In contrast to conventional methods of body weight measurements using weighing scales, a paradigm shift in use of AI technologies is evidenced as they facilitate accurate, time and labor-efficient measurement. Recently, the prediction of body weight non-invasively using images (computer vision technology), ANN, deep learning (DL) technologies and machine learning (ML) algorithms are also efficient and successful (Wang et al., 2021).

The use of depth images along with ML or DL algorithms for real-time BCS classification has been widely studied in cattle and can be adopted in small ruminant production. Further, Convolutional Neural Networks (CNN) DL model, computer vision and DL can accurately and precisely carry out BCS classification in small ruminants.

Growth evaluation using ANN to assess ADG has been used in sheep farming (Wang et al., 2024). In addition, computer vision and ML algorithms have wide scope in estimation and prediction of ADG in livestock (Wang et al., 2022). However, there is a scarcity of studies in small ruminants warranting future research efforts in these species.

Evaluation of feed conversion efficiency would assist in replacing less productive animals under heat stress conditions aiding in maintenance of productive, heat-tolerant flock. In that respect, evaluation of feed efficiency by monitoring feed intake in animals using AI tools have been efficient.

Training robust AI models requires large and well-annotated datasets. Likewise, in developing countries, the small ruminants are often reared in larger herd sizes making them the ideal species for developing AI-based growth evaluation models, as the size of the dataset required to train the models will be large and appropriate. Thus, AI tools are feasible for real-time, robust monitoring of growth traits in small ruminants. However, the limitations associated with the development and adoption of AI tools has been overlooked.

**Figure 5.**
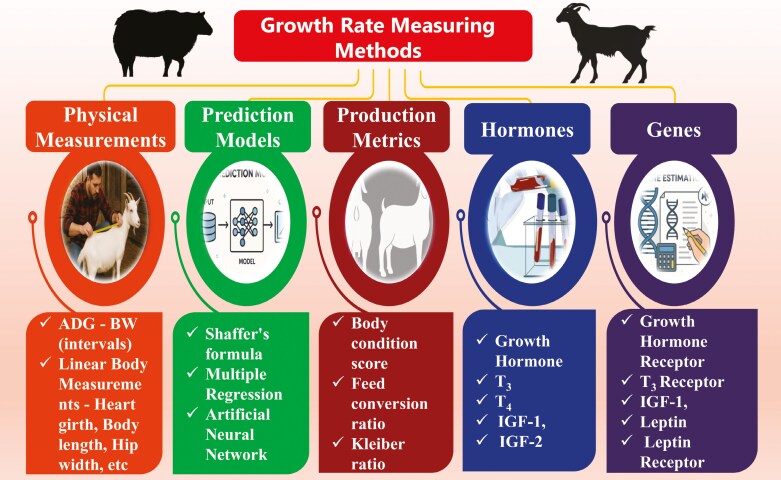
Growth assessing methods in small ruminants.

The development of AI tool for growth evaluation in small ruminants depends on various factors like species, breed, nutrition, age, stage of production, health, environment and so on, making it a demanding task to develop an universally acceptable model. There is limited research on development of a model that integrates these factors and thus a significant research gap exists in literature. Thus, future research efforts should be warranted in developing a user-friendly, field-deployable AI tool for predicting and evaluating growth in small ruminants, whilst accounting for variability due to other factors. Figure 5 illustrates various methods used to measure growth in small ruminants

## Strategies to Improve Growth Performance in Heat-Stressed Small Ruminants

### Shelter management

Due to intense heat periods during the day, animals reduce their grazing time and devote longer periods under shade. The most economical and efficient way to minimize intense thermal radiation is through provision of shade which can be either natural or artificial. In addition to shade, incorporating water-based cooling systems has proven effective in arid and humid climates, in lessening effects of heat stress on animals by inducing evaporative cooling either directly from the dermal surface of the animal or indirectly by the installation of a cooling pad or fans thereby alternating the microenvironment of the animal. The fan-and-fogging systems enhances air temperature and velocity within shelters, thereby reduces heat stress (Sejian et al., 2015).

Additionally, optimizing shelter design is crucial in alleviating heat stress concerns. Techniques such as installing double-wall structures with a 10 cm gap create a thermal barrier, minimizing conductive heat transfer into animal housing (Sejian et al., 2015). Further, mechanical ventilation provision with airspeed greater than 0.5 m/sec at closer proximity to the animals further lessens adverse effects of heat stress (Shaji et al., 2015).

Direct cooling methods, such as misting, spraying, and sprinkling systems, effectively reduce heat stress and improve animal welfare. Also, it can be used in conjunction with mechanical fans to enhance air circulation (Sejian et al., 2015). Further, cooling pads along with fan systems aids in regulating microenvironment (Shaji et al., 2015). Along with all these methods, optimal site selection for housing animals, agro-climatic zones and production systems-specific shed designs and implementation of rotational grazing strategies in shaded areas are essential prerequisites in limiting the impact of heat stress.

### Nutritional strategies

Nutritional interventions have emerged as critical adjunctive measures, directly influencing the animals’ ability to cope with elevated temperatures. Heat stress induces a cascade of metabolic changes, including increased energy expenditure (to sustain thermoregulatory mechanisms) and oxidative stress, resulting in increased nutritional demands, often unmet due to reduced feed intake, leading to deficiencies in essential micronutrients and altered metabolic processes (Rebez et al., 2024).

To combat the free radicals generated during heat stress, antioxidants such as vitamins C and E can be supplemented to stabilize the health of animal. Supplementation of Selenium-enriched *Saccharomyces cerevisiae* yeast is efficient in restoring antioxidant capacity and immune function of heat-stressed small ruminants. Similarly, high dietary levels of vitamin E and selenium improve feed intake, daily weight gain, and oxidative balance in heat-stressed animals. Modifying rations to optimize nutrient density is a key strategy that can be adopted by reducing dietary fiber content, incorporating high-quality fiber and protected fat supplements and adding feed additives (sodium bicarbonate, niacin, antioxidants, and yeast cultures) to enhance feed utilization (Al-Dawood, 2017).

Various feed supplements like betaine, vitamins C and E, selenium, herbs, probiotics, prebiotics and phytobiotics; protein sources such as fish meal, urea, and l-tyrosine; fat supplements including polyunsaturated fatty acids, *Ascophyllum nodosum*, olive pulp, and flaxseed; energy-dense feeds like grains and concentrates; fiber-rich options such as acacia, lucerne hay, and wheat straw; and essential mineral supplements can be tailored to meet the needs of heat-stressed animals (Rebez et al., 2024). Providing cool, clean, and adequate drinking water is crucial for managing heat stress.

### Genetic strategies

Management and nutrition strategies aid in temporary mitigation of heat stress, however, animal genetics can provide a long-term permanent solution for ensuring livestock production in the face of climate change (Sejian et al., 2018). Application of advanced biotechnological tools like Next Generation Sequencing technologies and statistical models, aids in identification of genetic biomarkers of heat stress. Particularly application of omics technologies like genomics, transcriptomics, proteomics, metabolomics, metagenomics and epigenomics are rapidly advancing and providing better knowledge on genetic architecture, thereby playing a vital role in identifying biomarkers for thermo-tolerance.

Genomic studies combined with statistical models such as Genome Wide Association Studies (GWAS) and Selection Signatures play a key role in uncovering the genetic variants that modulate the phenotypic responses to heat stress. For instance, the candidate genes *MYO5A, PRKG1, GSTCD,* and *RTN1* were identified to be the most significant SNPs associated with heat tolerance in the Egyptian sheep (Aboul-Naga et al., 2022). Additionally, GWAS analysis identified other heat tolerance-related SNPs in genes such as *PLCB1, STEAP3, KSR2, UNC13C, PEBP4,* and *GPAT2*. Likewise, selection signature have been annotated with several genes associated with heat stress/tolerance (FCGR1A, MDH1, UGP2, MYO1G, and HSPB3), immune response, cellular mechanisms associated with heat stress (Gaspa et al., 2024)

Researchers have also demonstrated the scope of omics approaches like transcriptomics, metagenomics, epigenetics, proteomics and metabolomics in identifying potential biomarkers for thermo-tolerance in livestock and crucial insights on animal’s adaptation mechanisms. A multidimensional approach of integrating omics technologies can offer a holistic framework providing a detailed understanding of the complex biological mechanisms governing adaptation mechanisms under heat stress (Sejian et al., 2019).

The biomarkers identified by integrating omics and statistical analysis approaches can provide a means to quantify heat stress responses specific to various adaptive mechanisms in small ruminants. These biomarkers can be integrated into breeding programs via Genomic Selection and Marker Assisted Selection, promoting selection and development of breeds more resilient to climate challenges (Sejian et al., 2018). Additionally, it is crucial to implement breeding and management practices specific to different agro-ecological zones.

**Figure 6.**
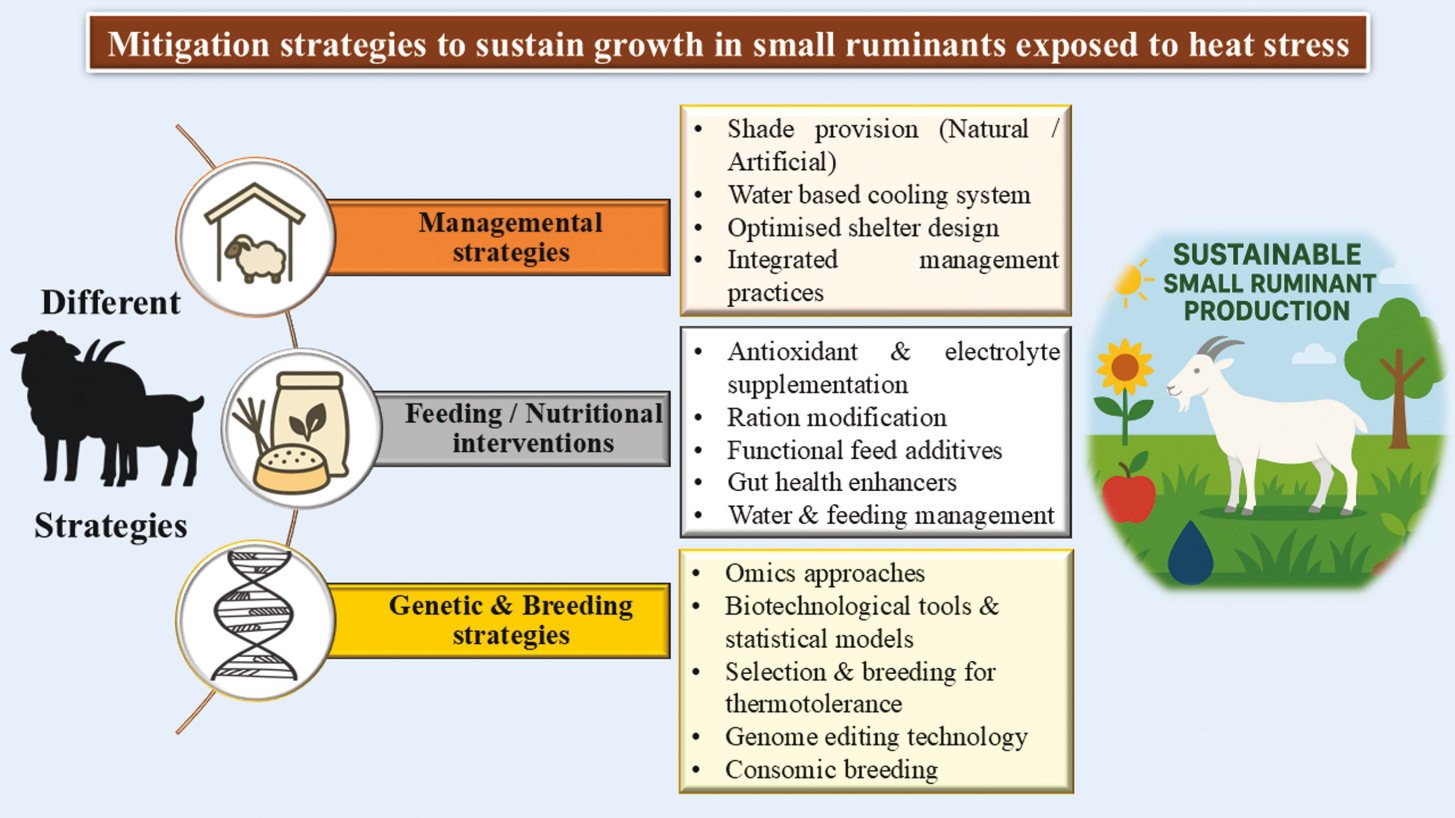
Mitigation strategies to sustain growth in small ruminants exposed to heat stress.

Further, genome editing technologies like CRISPR—Cas9, ZFN (Zinc Finger Nuclease), TALEN (Transcription Activator like Effector Nucleases) that are revolutionizing the field of biology (Ruan et al., 2017) can also be explored. For instance, CRISPR/Cas9 and TALEN has been employed in sheep and goats to modify genes, resulting in improved traits like increased muscle mass (Ni et al., 2014; Proudfoot et al., 2015). Similarly, expanding the application of these techniques to evaluate heat stress effects could open new avenues for improving the productivity in heat-stressed small ruminants. In addition, consomic breeding, a less commonly used genetic technique employed in plants and animals can also be explored as they facilitate mapping of quantitative trait loci (QTL). Experimental studies that combine consomic lines with next generation sequencing (NGS) approaches and statistical modeling can provide rapid and increased knowledge on the genes associated with tolerance or sensitivity to environmental stress (Collier et al., 2005). Such an approach can also be applied to improve climate resilience in small ruminants by introducing heat-tolerant chromosomal regions from resilient breeds, enabling the identification of stress resistance genes and enhancing productivity under harsh environmental conditions. Figure 6 depicts various mitigation strategies aimed at sustaining growth in heat-stressed small ruminants.

## Different Biomarkers for Assessing Heat Stress Impact on Growth Performance in Small Ruminants

Different biomarkers, both phenotypic and genotypic markers act as potential indicator of growth under heat stress conditions. Rectal temperature, skin temperature, respiration rate, sweating rate and FCR are reported to be the primary physical indicators of growth performance in heat-stressed small ruminants (Joy et al., 2020). Endocrine markers like T3, T4, cortisol, insulin and leptin are considered as essential hormones for assessing growth rate in heat-stressed animals (Habeeb et al., 2023). Further, serum cholesterol, glucose and electrolytes levels are lowered in heat-stressed animals and could be used for indirect assessment of growth potential in sheep and goats.

Apart from these phenotypic and serum biomarkers, several genetic biomarkers like *GH, GHR, LEP, LEPR, IGF-I*, *THR*, *BMP7, MSTN, STIL*, *SPP1, GJB2, GJB6, GJA3* and *BMP2* act as potential biomarkers for evaluating growth rate in heat-stressed small ruminants (Sejian et al., 2019; McManus et al., 2022). Thus, for assessing the impact of heat stress on growth rate in either sheep or goat the above discussed variables and genes can act as potential biomarkers.

## Conclusion

Small ruminant production ensures food and economic security of marginal farmers, especially in developing countries. Amidst the challenging climate, small ruminant production has been a vital component of both food and economic security, particularly in resource-limited and marginal agricultural regions. Among the various factors, heat stress was considered the most intriguing factor which negatively influences growth performance. This was attributed to the changes in feeding patterns and reduced nutrient absorption efficiency which results in reducing feed intake, weight gain, and overall productivity. Heat stress has a profound effect on growth performance of animals by impairing physiological and metabolic pathways, subsequently altering the both the endocrinology and expression patterns of genes that govern growth.

Heat stress alters the endocrine dynamics by reducing anabolic hormones responsible for promoting growth and development of muscles, and by increasing catabolic hormones, that catabolizes tissues and retards growth. Further, alteration in expression of the genes governing growth, thus these genes serve as valuable indicators of growth performance. However such heat stress driven endocrine and molecular responses governing growth performance are breed-specific, highlighting breed-specific adaptive responses to heat stress in small ruminants. The *GH, GHR, LEP, LEPR, IGF-I*, *THR*, *BMP7, MSTN, STIL*, *SPP1, GJB2, GJB6, GJA3* and *BMP2* are identified as potential biomarkers for evaluating growth rate in heat-stressed small ruminants. In contrast to conventional methods of body weight measurements using weighing scales, a paradigm shift in use of AI technologies is evidenced as they facilitate accurate, time and labor-efficient measurement.

Researchers have established that management and nutrition strategies could aid in temporary mitigation of heat stress, however, animal genetics through advanced genomic approaches integrating with AI tools applications can provide a long-term permanent solution for ensuring sustainable small ruminant production in the changing climate scenario. Thus, the future research in this line must focus on integrating AI and ML tools with advanced NGS technologies to identify more biomarkers which could help redefine the existing breeding program to produce more climate-resilient small ruminant breeds to count heat stress and sustain production.

## About the Authors

**Dr Silpa Mullakkalparambil Velayudhan**, M.V.Sc., Ph.D, is an Assistant Professor at Rajiv Gandhi Institute of Veterinary Education and Research, Puducherry, India. Dr. Silpa secured a merit scholarship for pursuing her Doctoral Research at Justus Liebig University, Giessen, Germany in Animal Genetics and Breeding. Her research primarily focuses on linking animal genetics with heat stress, its impact on animal production, adaptation and assesses thermo-tolerance, in goats and dairy cattle. She has been working on establishing the impact of heat stress on caprine skin using a holistic approach involving skin metagenomics, transcriptomics and epigenetics. She also worked on assessing the impact of heat stress in dairy cattle under field condition. Dr. Silpa has published 30 peer-reviewed publications 51 book chapters, edited 2 books, 21 invited papers, 43 conference abstracts and 14 NCBI sequence submissions. Attracted by her novel research, she has been an invited speaker in couple of international conferences. She also has nine best paper awards to her credit. She is a member in several professional societies in the field of climate change associated livestock production. She is a budding researcher with a lot of aspirations to do advanced research involving OMICS approaches to identify more biological markers for heat stress in livestock with the intention to develop new climate-resilient small ruminant breeds through marker assisted selection.

**Dr G. Ilavarasi** is an emerging researcher in the field of veterinary physiology with a focus on climate change and livestock production. She is currently an M.V.Sc Research Scholar at the Rajiv Gandhi Institute of Veterinary Education and Research (RIVER), Puducherry, India. Her primary research interest centers on the physiological, biochemical, nutritional, and immunological impacts of climate stressors on livestock, with a goal of developing sustainable adaptation and mitigation strategies. Dr. Ilavarasi has presented her work at numerous national and international scientific forums, with research contributions spanning thermo-tolerance traits, behavioral adaptation, molecular markers, and precision livestock farming. She has authored several abstracts and co-authored book chapters on climate-resilient livestock production, reflecting her strong commitment to advancing animal welfare in changing environmental conditions. Dr. Ilavarasi is a life member of the Indian Society for Sheep and Goat Production and Utilization (ISSGPU). Her practical experience spans clinical practice, laboratory diagnostics, and farm-level entrepreneurship, including a project on country chicken production. With growing expertise and international exposure at conferences in Turkey, Azerbaijan, and Cyprus, Dr. Ilavarasi G is poised to make significant contributions to climate-resilient animal agriculture.

**Dr Narasingam Balamurugan,** is the M.V.Sc., Scholar in Department of Animal Nutrition at Rajiv Gandhi Institute of Veterinary Education and Research, Puducherry, India. Currently, he is pursuing his research work at ICAR- ICAR-National Institute of Animal Nutrition and Physiology, Bangalore, India. His research focuses on the pathways of microbial metamorphosis and ruminal functional alterations in relation to heat stress and nutritional mitigation strategies. Dr Balamurugan was part of a team that developed a technology on “RIVER YES Mineral mixture for small ruminants of Puducherry” in 2024. He has secured Best Article award and consolation prize in the all India writing competition organized by Indian Veterinary Association, Kerala, India and eFeed. He was also awarded with Dr. C.M. Singh “Shalihotra” Pratibha Saman 2023 award, Dr. G.L. Jain National Award of Excellence in Poultry Science 2023, and Dr. V. Kurien Award of Excellence 2023 for his informative articles. Dr Balamurugan is the recipient of M.V.Sc scholarship by securing 3^rd^ rank in all India Entrance Examination for PG in 2023. He has several publications in peer-reviewed journals, book chapters and conference papers to his credit. He is serving as a Life member in Indian Society for Sheep and Goat Production and Utilization Society in India.

**Dr. R. Agnishwaran,** is the **M.V. Sc., Scholar** in the Department of Veterinary Public Health and Epidemiology at Rajiv Gandhi Institute of Veterinary Education and Research, Puducherry, India with a strong commitment to scientific research in the nexus of One Health, Climate Change and Public Health with a specific emphasis on Zoonotic disease monitoring and Food safety. Currently, he is working in innovative study on Bacteriophages as an effective and promising option to address antimicrobial resistance (AMR), which is a major worldwide health concern, with the goal of promoting healthier ecosystems and society. He has participated and presented papers in conference with the intention to disseminate knowledge and networking with researchers in his research field. He was awarded with Sri Awadhesh Kumar Singh Memorial Award for Best Popular Article Writing Competition held on the occasion of World Veterinary Day 2024 by Pashudhan praharee. He has secured Best Article awards in the all India writing competition organized by Indian Veterinary Association, Kerala and Chronicle of aquatic science journal. He has couple of peer-reviewed publications, book chapters and conference papers to his credit. He is a lifetime member of the Association of Public Health Veterinarians and the Indian Society for Sheep and Goat Production and Utilization.

**Dr. A Vanmathi** is a research Scholar pursuing her M.V.Sc in the Department of Veterinary Extension Education at Odisha University of Agriculture and Technology, Bhubaneswar, India. Hailing from Puducherry, India, she completed her Bachelor of Veterinary Science and Animal Husbandry from Rajiv Gandhi Institute of Veterinary Education and Research, Puducherry. Her current research focuses on “Knowledge level of goat farmers on climate change and its impact on goat production”, emphasizing climate-resilient livestock extension strategies. Dr.Vanmathi’s broader academic interest lies in climate change and livestock production, particularly the dissemination of lab-developed adaptive technologies to farming communities through effective veterinary extension frameworks. In recognition of her academic contributions, Dr.Vanmathi has been presented with the Young Researcher Award at the International Conference on Animal Science and Veterinary Medicine in 2023. She has authored and co-authored couple of research papers, book chapters, and conference papers on heat stress management, climate resilience in livestock, and sustainable livestock production systems. Dr.Vanmathi also completed a one-month externship program at Charles Sturt University, Australia, gaining exposure to advanced research facilities and molecular diagnostic techniques. She is a member of multiple professional organizations, including The New Zealand Veterinary Association and the American Meteorological Society, reflecting her active involvement in both veterinary and climate science communities.

**Dr Dona Mary Eldhose** is a postgraduate scholar in Animal Nutrition at the College of Veterinary and Animal Sciences, Pookode, Wayanad, Kerala. Her current research focuses on “**Evaluating the****impact of Ayurvedic residue supplementation on growth performance and nutrient utilization in ruminants”**, with the dual aim of reducing feed costs and enhancing immune function, ultimately contributing to sustainable livestock production. Dr. Dona Mary has established herself as a vibrant young scientist in animal nutrition and climate adaptation. Her scientific journey is enriched with national and international acclaims, including best paper presentation awards at prestigious conferences like ISSGPUCON and WVPA India. She has presented in multiple forums, delivering impactful presentations on topics such as nutritional strategies to mitigate methane emissions, the use of flaxseed in cattle diets, and the role of micronutrients in stress management. Dr. Dona Mary has also co-authored peer-reviewed articles and contributed to key book chapters. She completed an overseas externship at the University of Tuscia, Italy, where she gained practical experience in molecular techniques, feed analysis, sensor-based farm management, and Life Cycle Assessment (LCA) tools. Through her interdisciplinary research and global perspective, Dr. Dona Mary Eldhose is committed to building resilient livestock systems by integrating ethnoveterinary resources into modern nutritional science.

**Dr E. Angelin Shyona Darwin** is a promising young researcher and a postgraduate in the department of Livestock Products Technology at the West Bengal University of Animal and Fishery Sciences, Kolkata, India. With a strong academic background and deep interest in Livestock Products, her research focuses on “Fortification and Quality Evaluation of Meat Products with Phytochemicals.” Her scientific interests revolve around improving meat quality and functionality using natural bioactive compounds, with the aim of promoting sustainable and health-enhancing animal-derived foods. Dr. Angelin Shyona has presented nationally and internationally and has co-authored couple of peer-reviewed journal articles and book chapters. She completed an overseas externship at the University of Tuscia, Italy, gaining hands-on experience in advanced meat analysis techniques, sensor-based farm technologies, and international food safety protocols. Her academic journey is decorated with numerous awards, including best presentations and accolades in national and international conferences. She continues to contribute to the scientific community through active participation in webinars, workshops, and collaborative research. As a budding scientist, the career vision of Dr. Angelin Shyona Darwin is anchored in innovating sustainable livestock meat production enriched with functional phytochemicals to address public health and food security challenges.

**Dr. G. Kalaignazhal** is an M.V.Sc Scholar in the Department of Animal Genetics and Breeding at Orissa University of Agriculture and Technology, Bhubaneswar, India. She is an emerging researcher with a strong focus on addressing the impacts of climate change on livestock production. Her work focuses on the integration of animal breeding and genetics with climate change to identify and develop climate-resilient livestock breeds. Through her research driven strategies, she aims to advance sustainable livestock production systems to improve animal health, productivity, and adaptability in the face of ongoing climatic challenges. Dr. Kalaignazhal has been recognized with several prestigious awards for her academic excellence and research contributions, including the Chief Secretary’s Award for Academic Excellence and the Young Researcher Award, for her outstanding presentations at national and international conferences. She has contributed as both author and co-author of original research articles, review papers, and book chapters published in peer-reviewed journals. She enhanced her academic and research experience through a one-month externship program getting exposed to the advances in basic and fundamental research along with the exposure to the state-of-the-art research facilities both at the Charles Sturt University and the University of Melbourne, Australia.

**Dr. E. Binuni Rebez, M.V.Sc.,** is a budding and passionate researcher in Animal sciences. She holds a Master’s degree in Veterinary Physiology from Rajiv Gandhi Institute of Veterinary Education and Research (RIVER), Puducherry. Her academic and research interests are inclined towards building scientific knowledge in the discipline of Stress Physiology of livestock. Her Master’s work focused on identifying reliable biomarkers of heat stress and unraveled the efficacy of a herbal supplement in ameliorating heat stress in Kenguri sheep. Dr. Binuni has a prolific portfolio of scientific contributions, including several peer-reviewed journal articles, book chapters, and conference papers. She has earned numerous recognitions, notably for best presentations at national scientific forums and consecutive awards for best review article publications (2022–202, 2023–2024) from RIVER, Puducherry. Dr. Binuni has completed certified courses from Illinois, Stanford, IBM, University of Edinburgh and various open universities, equipping her with interdisciplinary skills in AI, molecular techniques, and animal welfare. She is motivated to enhance her skills and actively engages in webinars, workshops, and certified training programs to stay updated with advancements in her field. Her long-term goal centers on developing climate-resilient livestock systems, aligning with global goals of sustainable livestock production.

**Dr Sejian Veerasamy**, **M.V.Sc & PhD.,** is the Principal Scientist at ICAR-National Institute of Animal Nutrition and Physiology (NIANP), Bangalore, India. Previously he was serving as the Dean, Rajiv Gandhi Institute of Veterinary Education and Research, Puducherry. His current research focuses on identifying molecular markers for different environmental stresses in small ruminants with the primary focus to develop agro-ecological zone specific thermo-tolerant breeds. Dr Sejian is currently the Fellow of National Environmental Science Academy. Dr Sejian did his first post doctorate at The Ohio state University, USA. Dr Sejian was bestowed with the prestigious Endeavour Research Fellowship by the Australian Government to pursue post doctorate at The University of Queensland, Australia. For his outstanding contribution in animal science, Indian Council of Agricultural Research has bestowed him with the prestigious Lal Bahadur Shastri Outstanding Young Scientist Award in 2012. Further, Career360 an academic organization in India had selected Dr Sejian to be one of the top 10 scientists in India under environmental sciences category during 2017-18. Dr Sejian is also listed in world top 2% scientists for five consecutive years by Stanford University, USA during 2020, 2021, 2022, 2023 & 2024. In addition, Dr Sejian is also serving as Associate Editor in Elsevier’s Small Ruminants Research and Academic Editor in *PLOS* Climate. He has over 205 peer-reviewed publications, 158 book chapters, 320 invited/lead papers and 264 conference papers to his credit. He edited five springer books and one Elsevier book in his research field. He has also developed eight technologies and also has one patent granted. Corresponding author: drsejian@gmail.com.
